# Supplementary material for: The multilayer temporal network of public transport in Great Britain
Source: Sci Data. 2015 Jan 6;2:140056. doi: 10.1038/sdata.2014.56 (PMC4412150; doi:10.1038/sdata.2014.56)
Supplement: Supplementary Information [file sdata201456-s2.pdf]

The Multilayer Temporal Network of Public Transport in Great Britain

Riccardo Gallotti and Marc Barthelemy
Institut de Physique Théorique, CEA, CNRS-URA 2306, F-91191, Gif-sur-Yvette, France

SUPPLEMENTARY INFORMATION

In this Supplementary Information, we provide a statistical analysis of the quantities described in this dataset. The goals of this analysis is to check the limits of the data reliability and at the same time offering to future users a first insight into the characteristics of the Public Transport Network.

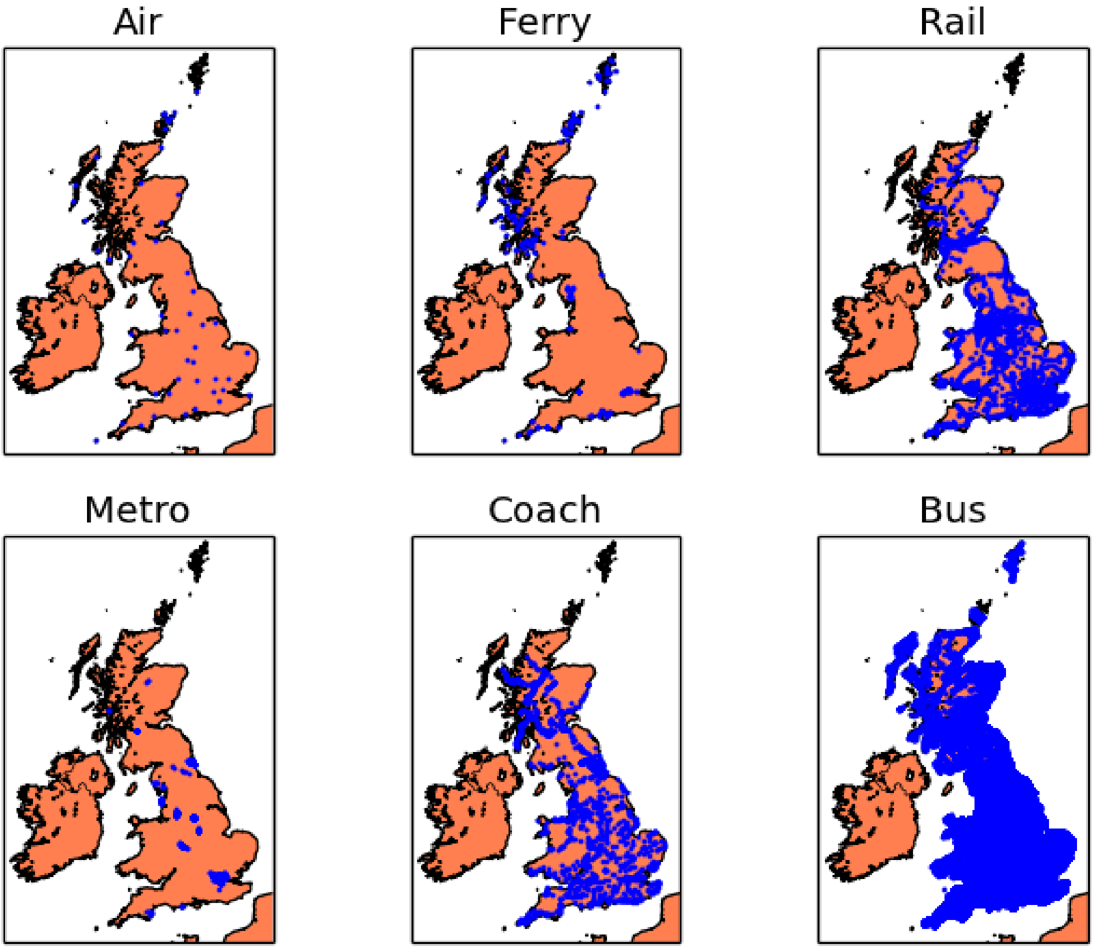

FIG. 1: **Location of the nodes of each layer.** The distribution of points matches the map of Great Britain. The Bus layer is covering the largest fraction of the island, while Ferry and Metro appear to be more concentrated in specific areas: the islands in the north of Scotland for the Ferry layer and the large urban areas of England for the Metro. The Coach, Rail and Air are more homogeneously spread, as they serve the purposes of inter-city connection across the whole country. (Figure produced with the Basemap python library).

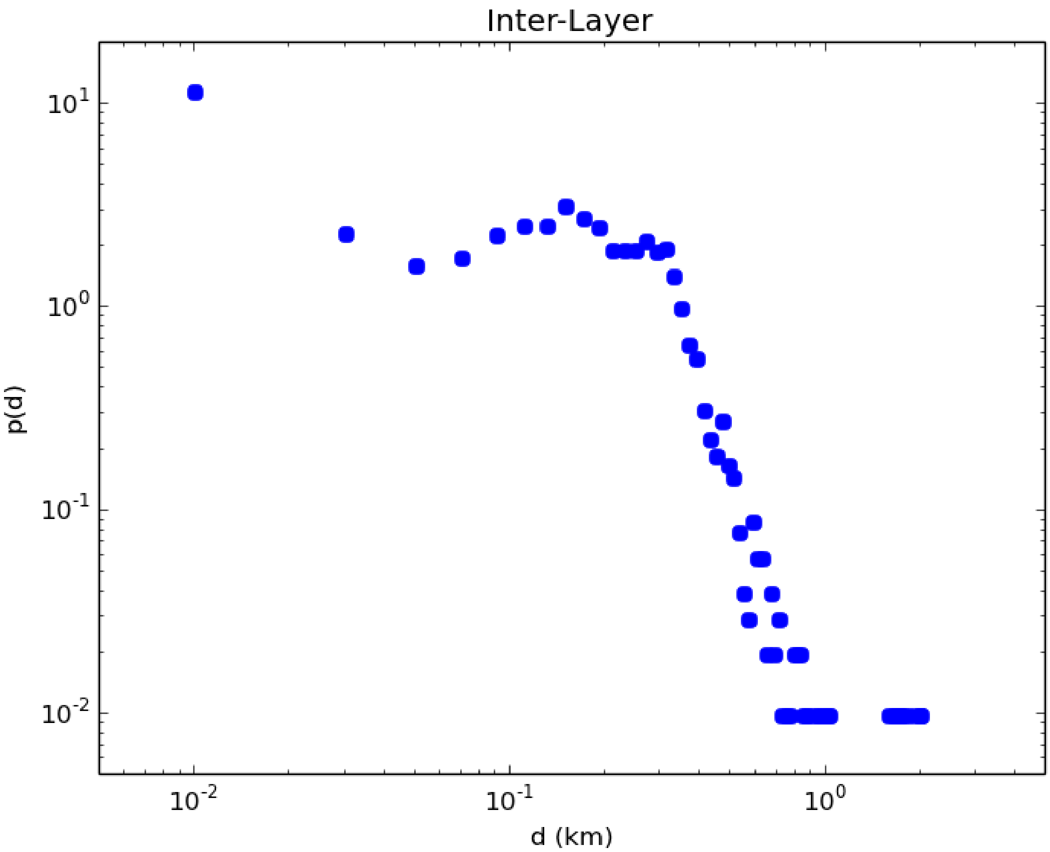

FIG. 2: **Inter-layer distance distribution.** The distribution is relatively uniform for values under 300 meters, and then quickly decays, having a maximum value of ≈2 km. These values are in agreement with the imposed maximal walking distance of 500 m imposed to low rank facilities and to the typical size of large facilities such as airports (for example, in order to reconstruct Heathrow airport we set a maximal distance of 3 km). We do not study the Inter-Layer travel time as it follows directly from the distance, the walking speed, and the minimal connection time.

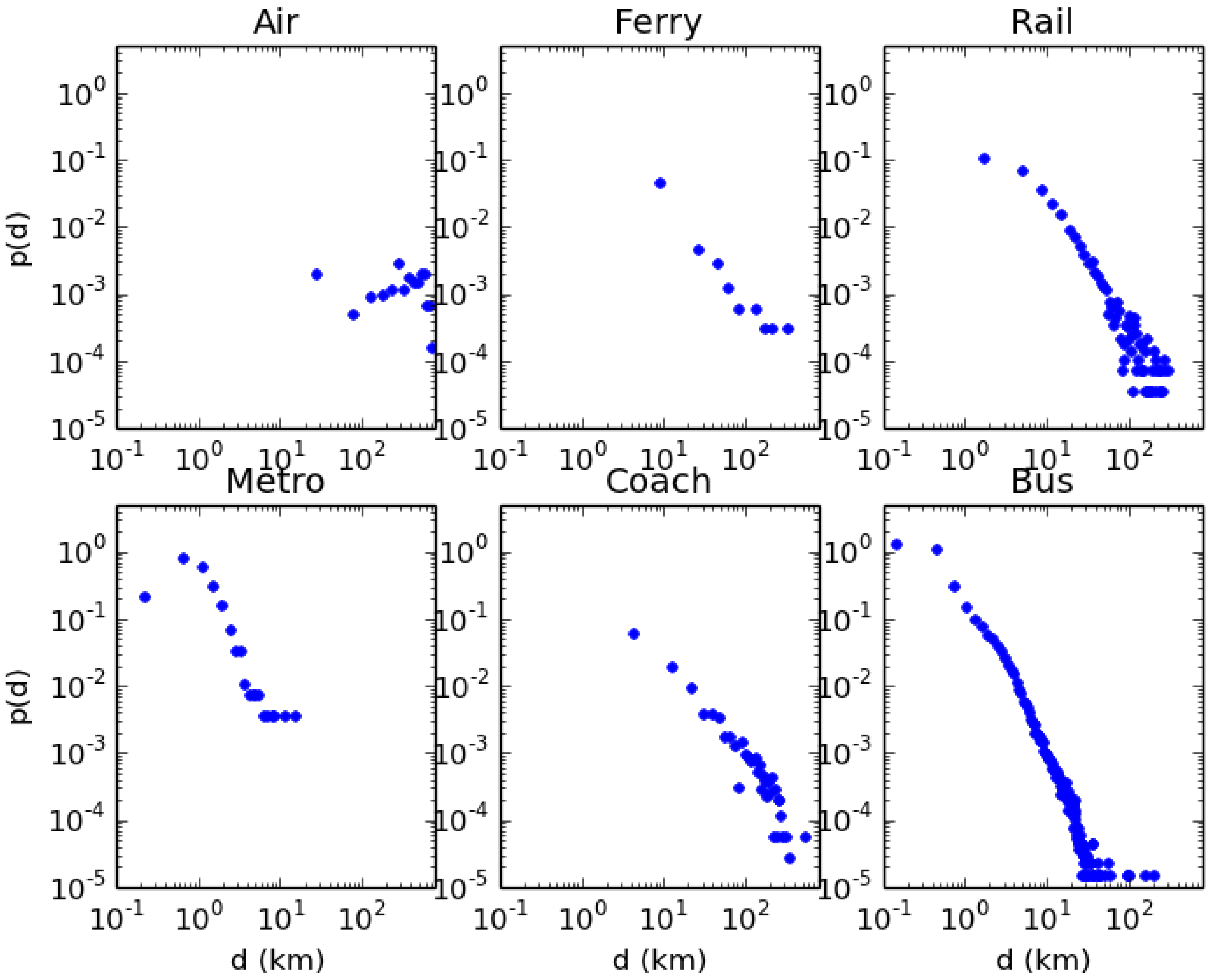

FIG. 3: **Intra-layer distance distribution.** Different layers have different characteristic distances. The largest part of Bus and Metro edges are short (<2 km), Rail edges appear above a minimal distance of ≈2 km, followed by Coach (≈5 km) Ferry (≈10 km). There is a significant fraction of Air edges under 100 km, mostly associated with low capacity or local flights between the Orkney Islands. Concerning the upper bound of the distribution, the longest distances are covered by flights. The Ferry, Rail and Coach layer appear all limited to maximal travels of ≈300 km. Metro edges are limited to the size of urban areas (≈10 km), while Bus can observed up to distances of order ≈50 km.

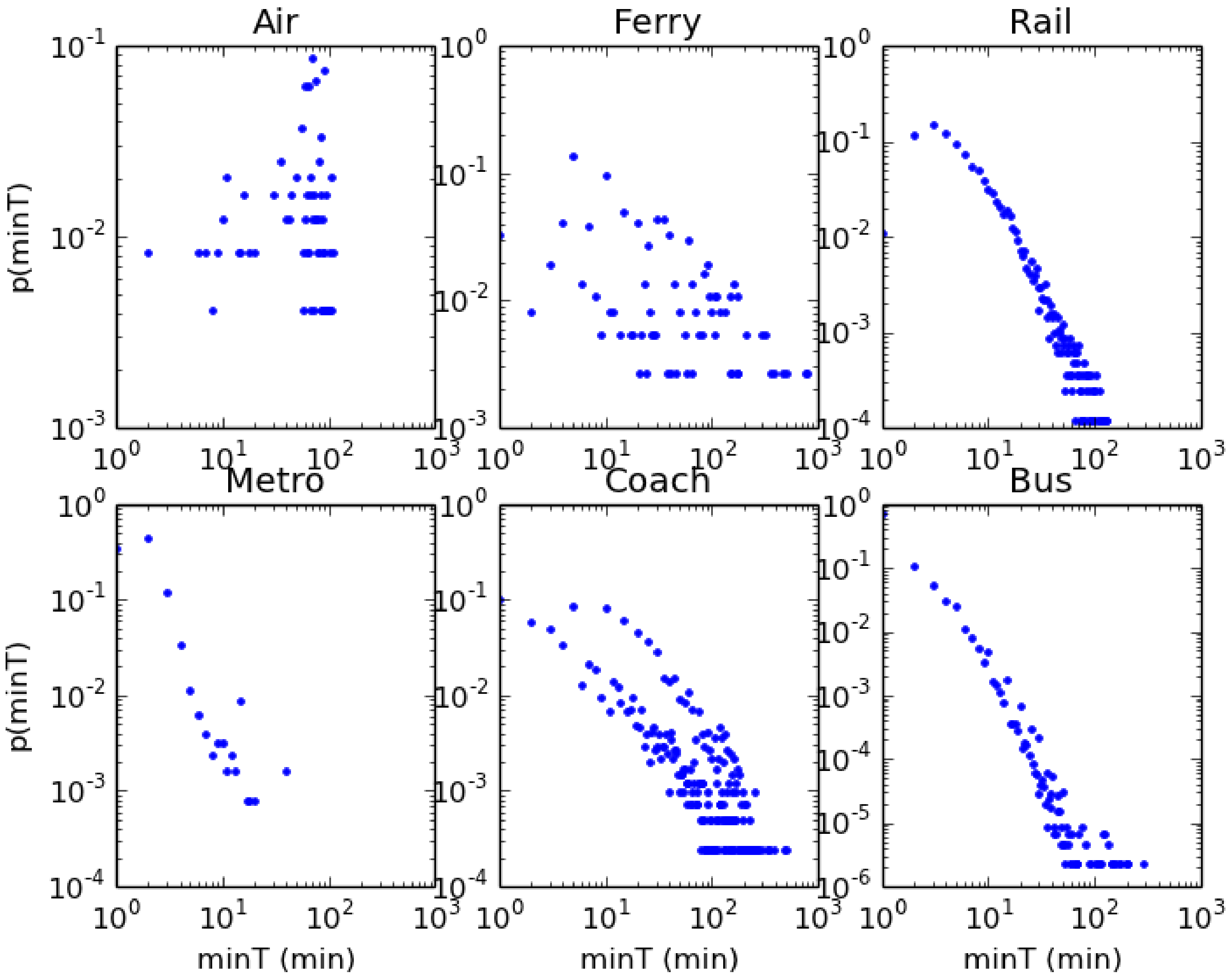

FIG. 4: **Intra-layer minimal traveltimes distribution.** Comparing traveltimes is less straightforward than comparing distances, as each layer has different typical speeds. In the Air, Rail and Bus the maximum traveltimes appear to be limited to ≈ 2h, while for Metro the value is much lower (≈20 min) and for Ferry and Coach higher. We observe a large number of minimal traveltimes of 1 minute in the Bus layer. The peculiar flight with a minimal time of 2 minutes has been identified as a small 9 seats aircraft covering a trajectory of 13 kms. The sparsity of the distributions, particularly noticeable for Air, Coach and Ferry is due to the preference of timetable designer for round number as, for instance, multiples of 5.

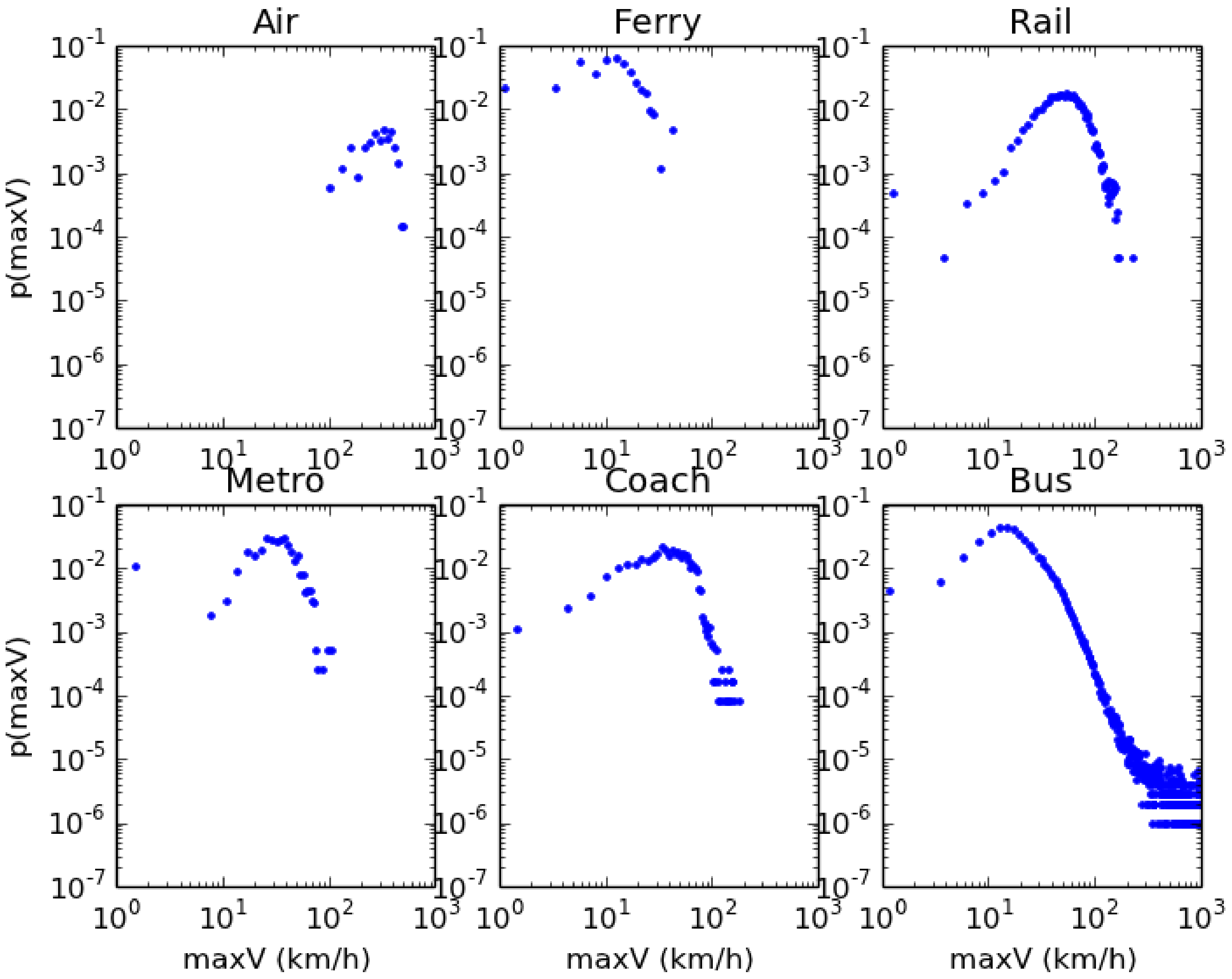

FIG. 5: **Distribution of the maximum speeds.** All the distributions present a maximum value consistent with what could be expected for the different transportation modes. Air: ≈ 350 km/h. Ferry: ≈ 15 km/h. Rail: ≈ 60 km/h. Metro: ≈ 30 km/h. Coach: ≈ 40 km/h. Bus: ≈ 15 km/h. We observe some cases impossible speeds for the Metro, Coach and Bus layers (with particularly extreme values in the latter: 7% of the bus edges have a maximum speed higher than 50km/h, 1% over 90km/h). As we see in figure 6, these extreme values are associated with errors in very short travel times.

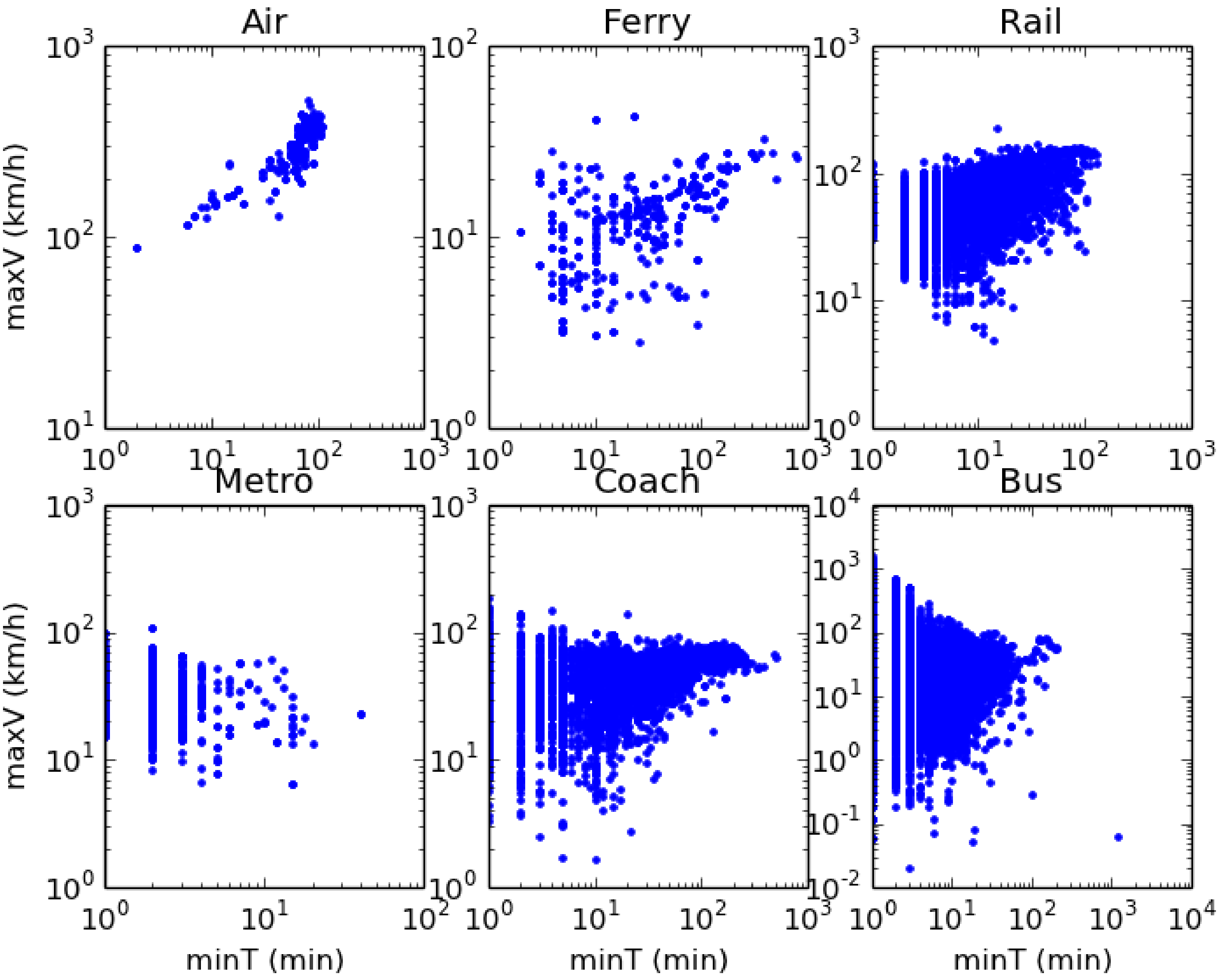

FIG. 6: **Maximum speed versus minimal traveltime.** As expected, we observe that for longer travel times, the speed increases. This behaviour is probably hidden in the sparsity of points for short times in the cases of the Metro, Coach and Bus layers. We see here that almost all high speed edges pointed out in figure 5 are associated to short traveltimes.

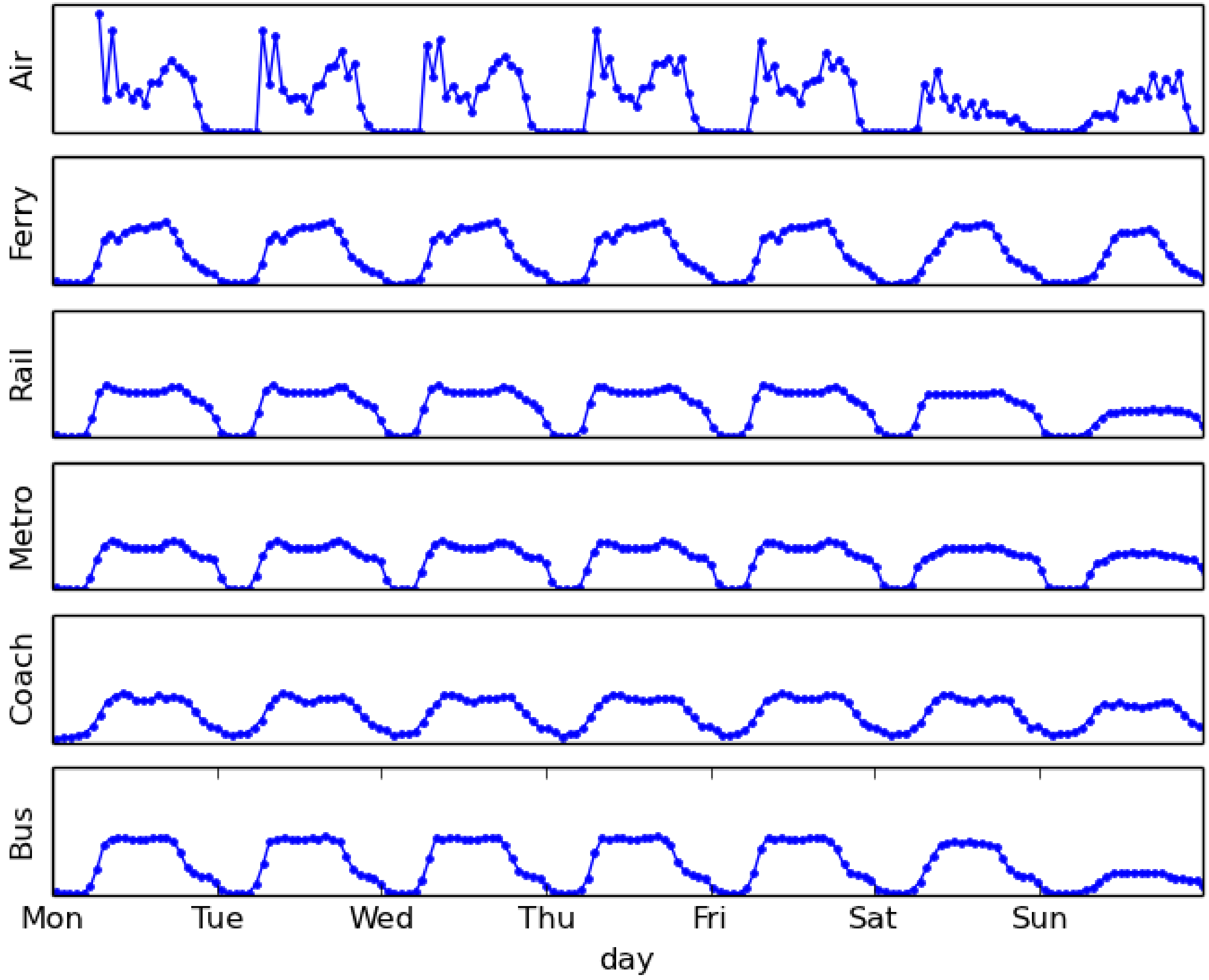

FIG. 7: **Distribution of the events times in the week.** As we see, events are spread during the days consistently with a periodical daily urban activity cycle. Weekdays are mostly similar to each other, while peculiar changes appear in schedules during the weekend.

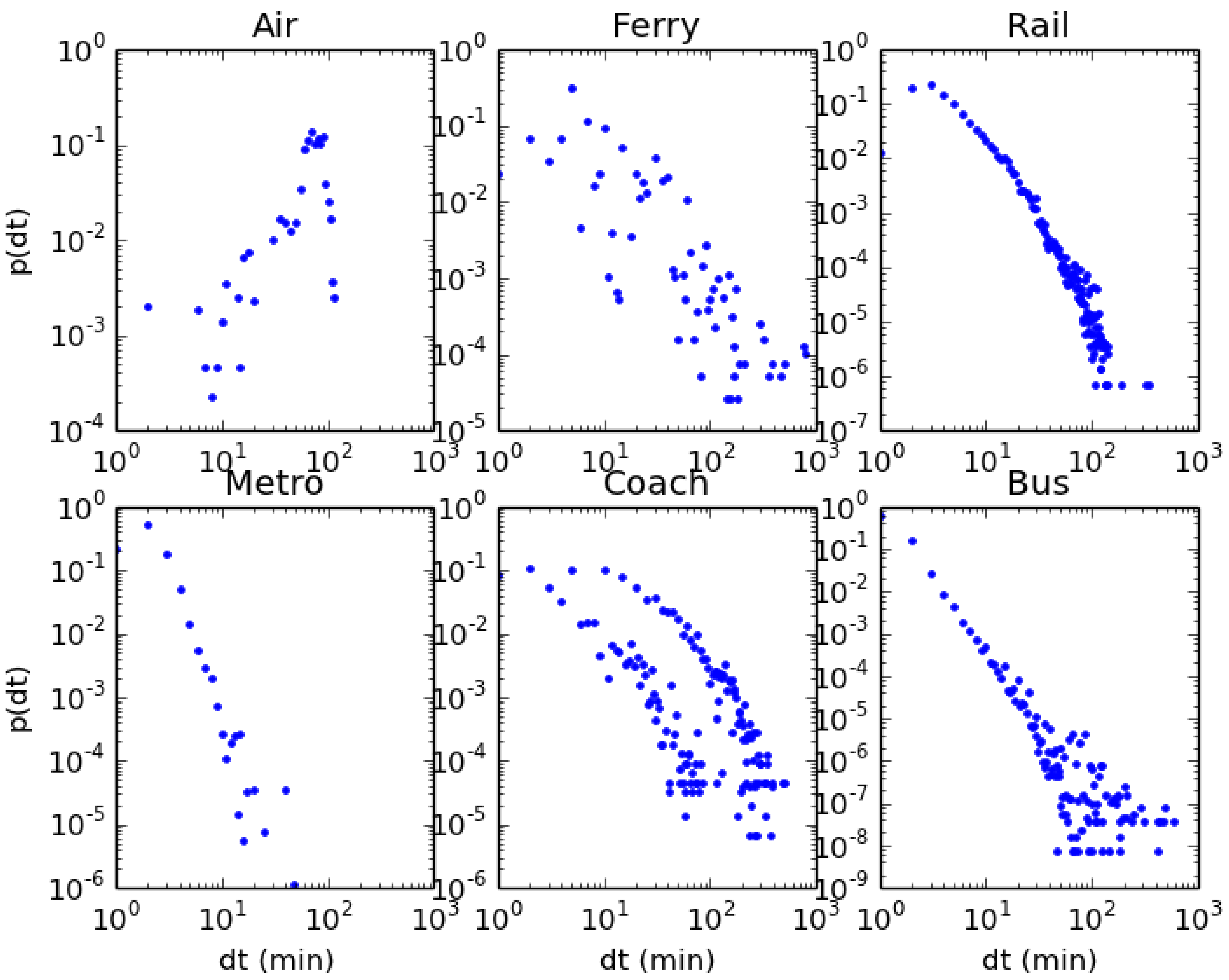

FIG. 8: **Distribution of the events' travel times.** Events' travel times present the same problems as minimal travel times. In some cases, events share the same time leading to too large or even impossible velocities. Indeed, in this case, travel times equal to zero have been kept in order to maintain the time causality of the sequences of events, implying that infinite speeds are a priori possible on single edges.
